# Nutrition therapy in critical illness: a review of the literature for clinicians

**DOI:** 10.1186/s13054-020-2739-4

**Published:** 2020-02-04

**Authors:** Kate J. Lambell, Oana A. Tatucu-Babet, Lee-anne Chapple, Dashiell Gantner, Emma J. Ridley

**Affiliations:** 1 Australian and New Zealand Intensive Care Research Centre, School of Public Health and Preventive Medicine, Monash University, Level 3, 555 St Kilda Rd, Melbourne, VIC 3004 Australia; 2 Nutrition Department, Alfred Health, Melbourne, Australia; 3 Department of Dietetics, Nutrition and Sport, La Trobe University, Melbourne, Australia; 4 Discipline of Acute Care Medicine, University of Adelaide, Adelaide, Australia; 5 Intensive Care Research, Royal Adelaide Hospital, Adelaide, Australia; 6 Intensive Care Unit, Alfred Health, Melbourne, Australia

**Keywords:** Critical illness, Intensive care, Nutrition therapy, Enteral nutrition, Parenteral nutrition

## Abstract

Nutrition therapy during critical illness has been a focus of recent research, with a rapid increase in publications accompanied by two updated international clinical guidelines. However, the translation of evidence into practice is challenging due to the continually evolving, often conflicting trial findings and guideline recommendations. This narrative review aims to provide a comprehensive synthesis and interpretation of the adult critical care nutrition literature, with a particular focus on continuing practice gaps and areas with new data, to assist clinicians in making practical, yet evidence-based decisions regarding nutrition management during the different stages of critical illness.

## Background

In recent years, there has been much interest in the role of nutrition therapy in critical illness with an increase in publications and two updated international clinical guidelines [1, 2]. However, trial findings and guideline recommendations continue to be conflicting, making the translation of evidence into practice challenging. Further, it is becoming evident that the stage of critical illness and individual factors such as body composition may be important when considering how individuals might respond to nutrition interventions [3, 4]. This narrative review aims to provide a summary and interpretation of the adult critical care nutrition literature, with a particular focus on continuing practice gaps and areas with new data, to help clinicians make practical, yet evidence-based decisions regarding nutrition management during critical illness.

## The metabolic response to critical illness and the role of nutrition therapy

It is recognised that ‘one-size fits all’ and ‘set and forget’ approaches to nutrition do not adequately address the complex metabolic, hormonal, and immunological changes that occur with critical illness [3, 5]. It is essential that clinicians understand these processes and the impact on nutrient metabolism [4]. In 1942, Cuthbertson described two distinct metabolic phases during acute illness—the ‘ebb’ or early shock phase, followed by the ‘flow’ or catabolic phase [6]. In brief, the ‘ebb’ phase is characterised by haemodynamic instability and hormonal changes (including insulin resistance) in order to prioritise the delivery of energy substrates to vital tissues [6, 7]. This survival mechanism results in endogenous glucose production as well as lower energy expenditure compared to pre-injury [4]. The ‘flow’ phase involves the breakdown of tissue (including lean muscle tissue) in order to provide substrates to cover the immediate needs for the ‘fight or flight’ response and to reduce the risk of bleeding and infection [4]. More recently, a third, anabolic recovery phase has been described [3]. It is during this recovery phase when resynthesis of lost tissue can take place and the body may be more metabolically able to process delivered nutrients [3, 4]. Currently, there is no known clinical marker to identify when an individual shifts from one phase of critical illness to another. For the purposes of this review which aims to provide practical recommendations, we have adapted the terminology from the 2019 European Society of Parenteral and Enteral Nutrition (ESPEN) critical care guideline to describe the different stages of critical illness: ICU day 1–2 (acute early phase), ICU day 3–7 (acute late phase), and after ICU day 7 (recovery phase) [2].

While it is considered that nutrition may be more physiologically available and hence more important in the later phase of illness, due to the average intensive care unit (ICU) length of stay (LOS), the majority of nutrition trials have provided nutrition interventions in the acute phases of illness (regardless of the intended trial intervention period). Traditionally, it was thought that aggressive nutrition in the early stages of critical illness may improve clinical outcomes. However, evidence from recent randomised controlled trials (RCTs) does not support this, finding no benefit or harm with early nutrition delivery [8–11]. An explanation for this may be because a substantial amount of energy was provided in a period of critical illness where energy expenditure is decreased and endogenous production is enhanced [4]. Specifically, harm was observed in The Early Parenteral Nutrition Completing Enteral Nutrition in Adult Critically ill Patients (EPaNIC) trial, the largest nutrition trial in critical illness [10]. In a study of 4640 mixed ICU patients (*n* = 2818 (61%) cardiac surgery patients) who were eligible to receive EN, late initiation of PN (started on day 8 of the ICU stay) led to an increase in the proportion of patients discharged alive and earlier from ICU and hospital (hazard ratio (HR) 1.06; 95% CI 1.00–1.13; *p* = 0.04 for both) when compared to PN commenced within 48 h of ICU admission [10]. Late initiation PN also led to a reduction in infectious complications (22.8% vs 26.2%, *p* = 0.008), cholestasis, duration of mechanical ventilation (MV), duration of renal replacement therapy, and health care costs [10]. Most recently, results from the largest enteral nutrition (EN) trial, The Augmented versus Routine approach to Giving Energy Trial (TARGET), support the theory that augmented energy delivery in the early phase of illness does not improve clinical outcomes compared to standard care [8]. This pragmatic prospective RCT of 3957 patients assessed 90-day mortality with augmented energy delivery (based on a predictive estimate of 1 ml/kg ideal body weight for height per day), compared to routine care [8]. Energy delivery was 50% higher in the intervention group (~ 30 kcal/kg ideal body weight/day) over the median 6-day nutrition delivery period (and approximated clinician estimated energy aims) but did not impact mortality or any secondary clinical outcomes [8]. However, it must be noted this study included a very ‘general’ (or unselected) population and that overfeeding may have occurred. Further post hoc work may increase the understanding and clinical implications of these results. Lack of benefit has also been observed with hypocaloric (low energy and adequate protein) and trophic (low energy and protein) feeding strategies compared to standard care, also provided early in critical illness and for short periods [9, 12]. The results of these trials support the hypothesis that for mixed ICU patients, nutrition interventions in the acute early and acute late phase of critical illness may not impact clinical outcomes and may cause harm in some groups. Therefore, less than 100% of energy expenditure should be targeted in this period due to endogenous glucose production. It remains unknown whether nutrition interventions continued for longer, impact functional recovery and quality of life [3].

## Guidelines for nutrition therapy in critical illness

There are currently four international clinical practice guidelines available to inform the nutrition management of critically ill patients [1, 2, 13, 14]. Table 1 summarises each guideline and outlines key recommendations and their level of supporting evidence.

**Table 1:** Key recommendations in clinical practice guidelines [1, 2, 13, 14]

Guideline	Basis of recommendation	Energy requirements	Protein requirements	Commencement of EN^a^	Commencement of PN
ASPEN/SCCM (2016)	Observational studies, RCTs, and consensus opinion from topic experts	Use IC (quality: very low) In the absence of IC use 25–30 kcal/kg/day (EC) Obesity: hypocaloric nutrition, 65-70% measured requirements by IC. If no IC, BMI 30–50 = 11–14 kcal/kg ABW/day; BMI > 50 = 22–25 kcal/kg IBW/day (EC)	1.2–2 g/kg/day (quality: very low) Obesity: high protein, BMI 30–40 = 2.0 g/kg IBW/day; BMI ≥ 40 = up to 2.5 g/kg IBW/day (EC)	Early EN (24–48 h) (quality: very low) Patients at low nutrition risk, well-nourished, and/or with low disease severity do not require specialised nutrition therapy over the first week in ICU (EC) Patients at high nutrition risk or severely malnourished, EN should advance to goal as quickly as tolerated over 24–48 h in (while monitoring for refeeding) (quality: very low)	Exclusive PN (when oral intake or EN contraindicated) for patients at low nutrition risk, withhold for the first 7 days (quality: very low) For patients at high nutrition risk or severely malnourished start PN as soon as possible (EC) Supplemental PN^b^ should be considered after 7–10 days if unable to meet > 60% of energy and protein requirements by EN (quality: moderate)
Canadian Clinical Practice Guidelines (2015)	RCTs and consensus opinion from topic experts	Nil	Nil	Early EN (within 24–48 h) (based on 16 level 2 studies^c^)	Exclusive PN (when oral intake or EN contraindicated) should be considered early in nutritionally high-risk patients (based on 6 level 2 studies^c^) For patients who are not malnourished, are tolerating some EN, or when PN is indicated for <10 days, low dose PN should be considered (based on 4 level 2 studies^c^) Supplemental PN^b^ should be assessed on case-by-case basis (based on 1 level 1 study and 7 level 2 studies^c^)
ESICM clinical practice guidelines (2017)	Observational studies, RCTs, and consensus opinion from topic experts	Nil	Nil	Early EN should be prescribed rather than delaying EN (low-quality evidence)	Nil
ESPEN (2019)	Observational studies, RCTs, and consensus opinion from topic experts	Use IC (grade B^d^) In the absence of IC use VO_2_ or VCO_2_ predictive equations (grade 0^d^) Obesity: if no IC, 20–25 kcal/kg ABW/day (grade 0^d^)	1.3 g/kg/day delivered progressively (grade 0^d^) Obesity: 1.3 g/kg ABW/day (grade 0^d^)	Early EN (within 48 h) (grade A^d^) Hypocaloric nutrition (< 70% of EE) in the early acute phase (ICU day 1–3) (grade B^d^) If using IC—isocaloric nutrition (80–100% EE) can be progressively implemented after day 3 (grade 0^d^) If using predictive equations—hypocaloric nutrition (< 70% of EE) for the first week (grade B^d^)	Exclusive PN (when oral intake or EN contraindicated) within 3–7 days (grade B^d^) For severely malnourished patients, consider early and progressive PN (grade 0^d^) Supplemental PN^b^ should be considered on a case-by-case basis (grade 0^d^)

*ABW* adjusted body weight, *ASPEN/SCCM* American Society of Parenteral and Enteral Nutrition/Society of Critical Care Medicine, *EC* expert consensus, *EE* energy expenditure, *EN* enteral nutrition, *ESICM* European Society of Intensive Care Medicine, *ESPEN* European Society of Parenteral and Enteral Nutrition, *IC* indirect calorimetry, *IBW* ideal body weight, *PN* parenteral nutrition, *RCTs* randomised controlled trials, *VO*_*2*_ oxygen consumption, *VCO*_*2*_ carbon dioxide production

^a^Commencement of EN in haemodynamically stable patients who are unable to maintain oral intake

^b^Supplemental PN—when all nutritional requirements are unable to be met by EN (i.e. due to intolerance, fasting)

^c^Canadian Clinical Practice Guidelines, level of bias for included RCTs: Level 1 = randomisation was concealed, outcome adjudication was blinded, and an intention to treat analysis was performed. Level 2 = if any one of the level 1 characteristics were unfulfilled

^d^ESPEN grade of recommendation: A = at least one high-quality meta-analysis, systematic review, or RCT; B = based on a body of evidence from well-conducted observational studies; 0 = case studies, expert opinion, or evidence extrapolated from high-quality systematic reviews or observational studies (recommendation refers to ‘can be aimed for’ rather than best practice)

## Energy in critical illness

Determination of energy requirements is one of the most significant challenges in critical illness and is of vital importance as prescribed targets are used to guide nutrition delivery. Predictive equations that estimate energy expenditure are the most commonly used method due to their ease of application but are often inaccurate compared to measured energy expenditure using indirect calorimetry [15]. Table 2 summarises why predictive equation estimates vary from measured energy expenditure [16, 17]. Importantly, inaccuracies increase at the extremes of weight, in the most severely unwell, and in older and more malnourished populations [16, 18]. Despite these failings, predictive equations continue to be widely used and are recommended in international clinical guidelines in the absence of indirect calorimetry [1, 2].

**Table 2:** Reasons equations for predicting energy expenditure lead to inaccurate results compared to measured energy expenditure [16, 17]

Equation factors	Patient and system factors
• Sample size of the original equation • The characteristics of the original development population (including age, body composition, and disease state) • The population characteristics in which the equation is used • The addition of commonly used ‘injury’ or ‘stress’ factors • Using an adjusted body weight with the equation	• Individual patient heterogeneity in the metabolic response to critical illness • Differing body composition (fat-free mass and fat mass) • Changes in medical management over time in elements that impact metabolism (like surgery, pain, and sedation practices)

### Estimating energy expenditure via VO_2_ and VCO_2_

Due to the persistent inaccuracies associated with the use of predictive equations, other methods (many of which have existed for some time) have been recently recommended in the 2019 ESPEN critical care guideline in the absence of indirect calorimetry [2]. Resting energy expenditure (REE) can be estimated via VCO_2_ (carbon dioxide production) from the ventilator and the rewritten Weir formula (REE = VCO_2_ × 8.19) or using VO_2_ (oxygen consumption) from a pulmonary artery catheter via the Fick method [19–22]. A recent study in 84 critically ill patients reported a higher level of agreement between energy requirements estimated by the VCO_2_ method and measured REE compared to other predictive equations [20]. There are methodological limitations to note with this method: an assumed normal respiratory quotient (RQ) of 0.85 is used, which is the RQ of most nutritional products (with RQ = VCO_2_/VO_2_, normally ranging between 0.67 and 1.2 depending on the proportion of carbohydrate, fat, and protein being combusted) [23]. However, in critical illness, RQ may also be influenced by endogenous glucose production and by periods of hypo- and hyper-ventilation, and is likely to fluctuate between populations [19, 20].

### Measuring energy expenditure in the critically ill—indirect calorimetry

Indirect calorimetry allows for the measurement of VO_2_ and VCO_2_ through the ventilator and is the gold standard method for measuring REE in critical illness when ideal test conditions are implemented [24]. Both the European (ESPEN) and American (ASPEN/SCCM) clinical practice guidelines recommend the use of indirect calorimetry to measure energy expenditure (Table 1) [1, 2].

Despite the guideline recommendations, only three single-centre RCTs have investigated the impact of delivering energy according to a measured energy expenditure (via indirect calorimetry) compared to energy delivery using a 25-kcal/kg/day estimate (standard care) on clinical outcomes. The first, published in 2011, included 130 patients and observed a trend towards reduced hospital mortality (primary outcome) in the intervention group using intention to treat (ITT) analysis (*n* = 21/65, 32.3%, vs 31/65, 47.7%, *p* = 0.058) [25]. However, infectious complications (*n* = 37 vs 20, *p =* 0.05) and mean (± standard deviation) duration of MV (16.1 ± 14.7 vs 10.5 ± 8.3 days, *p* = 0.03) and ICU LOS (17.2 ± 14.6 vs 11.7 ± 8.4 days, *p* = 0.04) were increased in the intervention group compared to standard care [25]. In a more recent and slightly larger trial of 203 patients, no differences were observed in the primary outcome (self-reported physical component summary score of SF-36 at 6 months) between intervention and control in the ITT analysis (*n* = 199, 22.9 vs 23.0, *p* = 0.99, respectively) or in any clinically important secondary outcomes [11]. However, in a post hoc analysis, a longer median (interquartile range) ICU LOS was observed in the intervention group (8 (5–25) vs 7 (4–12) days, *p* = 0.03) [11]. Lastly, in a pilot study (*n* = 40), no statistically significant differences were observed between groups in the primary outcome of change in bioelectrical impedance phase angle (related to nutritional status and prognosis) from baseline to ICU discharge [26]. However, a declining trend in mean phase angle was observed in the standard care group (3.31 ± 1.34° to 2.95 ± 1.15°, *p* = 0.077), and a significantly shorter ICU LOS was reported in the intervention versus the standard care group (13 ± 8 vs 24 ± 20 days, *p* < 0.05) [26].

Consistently across all three RCTs, indirect calorimetry was feasible and energy targets were more closely met when using indirect calorimetry in place of fixed-energy prescription. Methodological characteristics must be noted in interpreting these results; all studies were unblinded and single-centre in design and were likely underpowered to demonstrate true differences in clinical and functional recovery outcomes. Further, these studies aimed to meet 100% of indirect calorimetry targets early in the ICU admission, which recent evidence suggests is not beneficial, and there was limited investigation into high-risk subgroups in which indirect calorimetry may have avoided harm by under- or overfeeding (i.e. obesity). Despite this, these studies do not suggest that indirect calorimetry to guide energy delivery is superior to using predictive equations with regard to improving clinical outcomes.

### Measurement or estimation of energy expenditure?

Regardless if energy expenditure is measured or estimated, there is no consensus on how much energy should be provided. Based on current evidence, the most significant benefit of using indirect calorimetry is to personalise energy prescription and avoid under- or overdelivery of energy across the different phases of critical illness. For this reason, it is the opinion of the authors that if indirect calorimetry is available, it should be used primarily in patients where the clinicians are concerned about under- or overestimating energy needs (i.e. obese and underweight individuals) [27]. When used, clinicians should aim for high-quality tests by reaching a steady test state (defined as a variation in VO_2_ and VCO_2_ less than 10% over five consecutive minutes), conduct tests for ≥ 30 min, and repeat tests at least weekly (or more frequently if clinically indicated) [24].

For the majority of clinicians, current practice will continue to include the use of a predictive equation for estimation of energy needs. Clinicians must be aware that accurate estimation of energy expenditure with a predictive equation requires considerable knowledge of the underlying patient condition, the factors that alter the metabolic response to illness, and the limitations of the equation being used. It is also important to consider that delivery of calories to meet measured or estimated energy expenditure may not equate to what should be provided to improve outcomes. This may be particularly relevant in the acute early phase of critical illness where endogenous substrate mobilisation provides a substantial portion of the energy requirement and insulin resistance occurs, and therefore, a conservative energy target should be the aim [28]. Energy prescription and energy delivery (including non-nutritional sources such as dextrose and propofol) should be regularly reviewed in the context of the patient’s clinical condition and metabolic phase to prevent considerable under- or overfeeding [29].

## Protein in critical illness

In states of stress, such as in critical illness, the synthesis of acute phase proteins and those involved in immune function increase to support recovery [30]. Rapid and significant loss of skeletal muscle mass occurs to provide precursor amino acids to aid this process [31]. Despite a lack of definitive evidence, clinical guidelines recommend protein delivery of between 1.2 and 2 g/kg/day (Table 1) based on the assumption that like energy, delivery of adequate protein will attenuate skeletal muscle wasting and improve clinical outcomes. The ASPEN/SCCM guidelines also make recommendations for higher protein provision in specific clinical conditions (i.e. burns, obesity, and multi-trauma), which again are based on limited, primarily observational data and expert opinion [1]. The variation in the clinical guideline recommendations for protein delivery reflects the lack of good quality trials investigating the role of protein provision on clinical outcomes.

### Protein delivery and clinical outcomes

Higher protein provision has been associated with improved survival in a number of observational studies [32–36]. Conversely, higher protein delivery during ICU admission has led to increased urea production and has been associated with increased muscle wasting in a small observational study [10, 11, 31, 37].

In RCTs aiming to compare high versus lower protein delivery in critical illness, no benefit has been shown with an increased protein dose, although most have been underpowered to demonstrate an effect on clinical outcomes [11, 37–39]. The largest RCT (*n* = 474) investigating intravenous protein provided at a dose of up to 100 g/day compared to standard care found no impact on the primary outcome of renal dysfunction [37]. A smaller RCT compared intravenous protein at a dose of either 0.8 g/kg (*n* = 60) or 1.2 g/kg (*n* = 59) delivered over ten days while controlling for energy intake [38]. While there was no difference in the primary outcome of handgrip strength, the group who received the higher protein dose had less fatigue and higher forearm thickness (using ultrasound) at day 7 [38]. However, these findings may be impacted by unadjusted confounders and must be interpreted with caution [40].

Timing of protein delivery may also influence clinical outcomes. Two observational studies have reported increased survival with early increased protein delivery (day 3–4) [32, 33]. In the largest study (*n* = 2253), early protein delivery (> 0.7 g/kg/day versus ≤ 0.7 g/kg/day) was associated with increased survival (adjusted HR 0.83, 95% CI 0.71–0.97, *p* = 0.017) [33]. Contrary to these findings, in a post hoc secondary analysis of the EPaNIC trial, a cumulative protein dose, rather than the cumulative glucose dose, early during ICU stay was associated with delayed ICU discharge [41]. Further, a single-centre retrospective cohort study (*n* = 455) reported a lower protein intake (< 0.8 g/kg/day) before day 3 and high protein intake (> 0.8 g/kg/day) after day 3 was associated with lower 6-month mortality (adjusted HR 0.609; 95% CI 0.480–0.772, *p* < 0.001) compared to patients with overall high protein intake [42]. Prospective, randomised data is required to inform the most appropriate amount and timing of protein to deliver to critically ill patients. Adequately powered RCTs are urgently needed to better understand the impact of both protein dose and timing on clinical outcomes in critical illness. Such trials should ideally control for energy delivery, by ensuring it is consistent across both the intervention and control groups.

## How much energy and protein do patients get in clinical practice?

One of the most important pieces of information that clinicians should consider is that patients do not receive the energy and protein dose that is prescribed. In a recent retrospective observational study of 17,524 patients, the mean ± standard deviation energy and protein received was 56 ± 30% and 52 ± 30% of the intended aim, respectively [43]. This has consistently been shown across different time periods and geographical regions [44]. The reasons for this are multifactorial, including interruptions to EN for procedures, delayed initiation of nutrition, and gastrointestinal intolerance [45].

## What energy and protein targets should clinicians aim for?

**Fig. 1:**
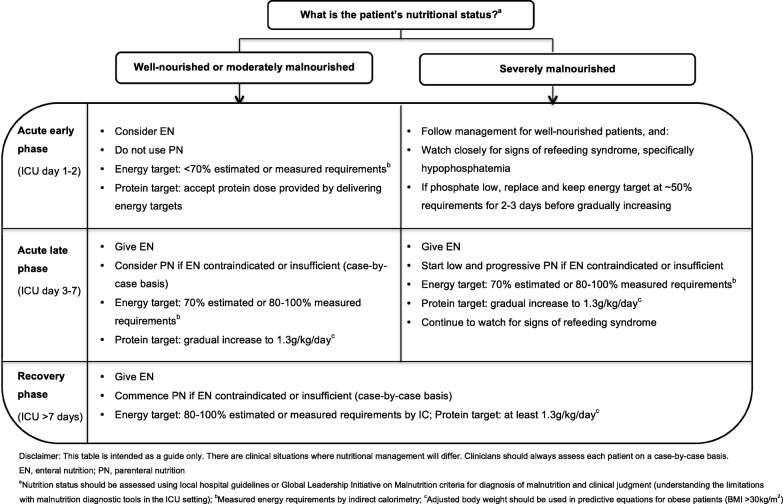
Recommendations for nutritional management by nutritional status and phase of critical illness

In light of the current evidence, the authors support the gradual introduction of nutrition therapy during the acute phases of critical illness, with energy and protein targets outlined in Fig. 1. In patients who are ‘at risk’ of refeeding syndrome, it is crucial that nutrition therapy is introduced slowly, and electrolytes are monitored closely and replaced as necessary [46]. If hypophosphatemia is present (e.g. < 0.65 mmol/l) in the first few days after starting nutrition therapy, then energy delivery should be restricted to ~ 50% requirements for 2–3 days [47].

## Enteral nutrition

### When to start?

Early provision of EN (within 48 h of ICU admission) in patients who are mechanically ventilated is an established standard of care and supported by all clinical guidelines [1, 2, 13, 14].

### How should EN be delivered?

The most common method of delivering EN in ICU is via a gastric tube, with a continuous hourly infusion. However, this continuous supply of nutrients does not mimic normal volitional intake which is most commonly in the form of boluses followed by periods of fasting. Recently, it has been proposed that bolus (intermittent) feeding may be more physiologic and therefore superior to continuous feeding [48]. A systematic review was conducted as part of the recent ESPEN guidelines to investigate whether bolus EN has an advantage over continuously administered EN [2]. Including 5 small prospective studies and 236 patients, a significant reduction in diarrhoea was observed with continuous versus bolus administration of EN (RR 0.42, 95% CI 0.19–0.91, *p* = 0.03) [2]. No differences in gastric residual volume, rates of aspiration, or pneumonia were observed. It has also been suggested that muscle protein synthesis may be improved when EN is delivered via a bolus when compared to continuous delivery, and a phase II multicentre RCT has recently completed recruitment to investigate this question (ClinicalTrials.gov NCT02358512) [5, 48]. Moving from continuous to bolus delivery of EN in the ICU is a significant change to practice in most countries, which would require a variation in feeding protocols and extensive education of clinical staff. Due to the considerable practice change associated, until definitive evidence is available to support one method of delivery over the other, it is reasonable that clinicians continue to deliver EN via a continuous infusion.

### EN delivery—an ongoing challenge

International guidelines are unanimous in favouring EN delivery into the stomach or small bowel over parenteral nutrition (PN) [1, 2]. Due to continued and consistent recommendations to meet energy requirements over many years, many strategies to ‘optimise’ EN delivery closer to predicted targets have been tested, including the use of evidence-based feeding protocols, small bowel feeding tubes, prokinetic drugs, and increase of the acceptable gastric residual volume [49–58]. Guideline recommendations to maximise EN delivery are summarised in Table 3. Despite the implementation of such interventions, these trials have observed modest to no increase in nutrition delivery and none have demonstrated a beneficial effect on clinical outcomes, potentially related to the disconnect between ‘delivery’ and ‘utilisation’ of nutrients.

**Table 3:** Guideline recommendations for strategies to improve EN delivery [1, 2, 13, 14]

Strategy	Evidenced-based feeding protocol	GRV (minimum 500 ml cut-off)	Appropriate and timely use of prokinetics for EN intolerance	Post-pyloric tubes for EN intolerance
ASPEN/SCCM (2016)	Use an EN protocol (designed and implemented to increase the overall percentage of goal energy delivered) (quality: moderate to high)	Do not use GRVs as part of routine care to monitor ICU patients on EN If GRVs are used, use 500ml cut-off (quality: low)	Use metoclopramide or erythromycin where indicated (quality: low)	Nil
Canadian Clinical Practice Guidelines (2015)	Use an EN protocol (that details strategies to improve delivery of EN) (based on 2 level 2 studies and 3 cluster RCTs^a^)	Use GRV of 250–500 ml every 4–6 h (based on 3 level 2 studies^a^)	Use metoclopramide where indicated (based on 1 level 1 study and 5 level 2 studies^a^)	Use post-pyloric tubes for patients at high risk for intolerance to EN or aspiration (based on 16 level 2 studies^a^)
ESPEN (2019)	Nil	EN should only be delayed when GRV is > 500 ml/6 h (grade 0^b^)	Use IV erythromycin as a first line therapy (grade of recommendation B^b^) or use IV metaclopramide or combination therapy (grade 0^b^) Alternatively, combination therapy (IV metoclopramide and erythromycin) (grade 0^b^)	Use post-pyloric feeding for EN intolerance not resolved with prokinetics (grade B^b^)

*ASPEN/SCCM* American Society of Parenteral and Enteral Nutrition/Society of Critical Care Medicine, *EC* expert consensus, *EN* enteral nutrition, *ESICM* European Society of Intensive Care Medicine, *ESPEN* European Society of Parenteral and Enteral Nutrition, *GRV* gastric residual volume, *ICU* intensive care unit, *IV* intravenous therapy

^a^Canadian Clinical Practice Guidelines, level of bias for included RCTs: Level 1 = randomisation was concealed, outcome adjudication was blinded, and an intention to treat analysis was performed. Level 2 = if any one of the forementioned characteristics was unfulfilled

^b^ESPEN grade of recommendation: A = at least one high-quality meta-analysis, systematic review, or RCT; B = body of evidence from well-conducted observational studies; 0 = case studies, expert opinion, or evidence extrapolated from high-quality systematic reviews or observational studies (recommendation refers to ‘can be aimed for’ rather than best practice)

## Parenteral nutrition

### When to start?

PN is indicated when the delivery of nutrients via the gastrointestinal tract is contraindicated or insufficient. PN can be provided either as a full source of nutrition (exclusive PN) or as an additional nutrition source when full requirements are not able to be met by oral intake or EN (supplemental PN). Recent RCT evidence has indicated there are no differences in clinical outcomes, including mortality and infective complications, when PN is provided versus EN in a modern day ICU setting and when energy provided is comparable in both groups [59, 60]. Guideline recommendations for when to commence PN differ and are outlined in Table 1. Due to the potential harm with early PN, it is the opinion of the authors that if oral intake or EN is contraindicated, then PN should only be considered between ICU days 3 and 7 and that supplemental PN be considered on an individual case-by-case basis (Fig. 1).

## Body composition analysis

The measurement of weight and muscularity is important in the assessment of nutrition status and monitoring the effectiveness of nutrition interventions [61]. However, due to the extreme fluid shifts that critically ill patients experience, measured weight and/or muscularity assessed by traditional bedside methods (e.g., subjective physical assessment, mid-arm muscle circumference) may be inaccurate in this patient population [62–64]. Table 4 summarises the emerging tools for assessment of muscularity in the ICU setting: computed tomography image analysis, bioimpedance analysis, and ultrasound. Currently, these methods for assessing muscle mass and quality are mostly limited to research [64–66]. There is an essential need to evaluate which bedside tools can accurately measure muscle mass, and identify those individuals with lower than normal muscularity, as well as to better understand the clinical importance of changes in muscle health and the interface with nutrition interventions in critical illness.

**Table 4:** Methodologies for assessment of skeletal muscle in ICU [64–66]

Method	Measurement	Details	Benefits	Limitations
CT image analysis at the abdominal (L3) area	• Muscle CSA (cm^2^) • Muscle quality (density) (Hounsfield units)	• Specialised software can be used to measure muscle area and density using a CT slice at L3 • Quantification of muscle CSA at L3 is highly correlated to whole body muscle (using scans performed for clinical purposes)	• Provides specific and precise results • Published cut-off values to identify patients with lower than normal muscularity	• Limited for use in patients who have had a CT at L3 area • Specialist training and time required for analysis
Bioimpedance analysis (multi-frequency or spectroscopy)	• Fat-free mass (kg) • Phase angle (50 kHz)	• Involves application of a weak current at differing frequencies, through electrodes placed on the hands and feet • Total body water, percentage body fat, and fat-free mass are estimated via regression equations (with assumed constants for estimating intra- and extracellular water) • Raw data such as phase angle (which is independent of weight and related to cellular health) may be a predictor of outcome in critically ill populations	• Easy and quick to use • Safe (no radiation involved)	• Fat-free mass estimates are not likely to be reliable in critically ill patients who experience significant fluid shifts • Positioning (separation in limbs) and electrode placement may be challenging in some ICU patients
Ultrasound	• Muscle thickness (cm) • Muscle CSA (cm^2^) • Muscle quality (echogenicity)	• Muscle thickness and CSA can be measured at different sites (i.e. quadriceps, upper arm) • Muscle quality can also be assessed using specialist software	• Readily available in most ICUs • Easy, safe, and quick to use	• No consensus on the ideal sites to predict whole body muscle or to monitor changes over time • Role of oedema on measurements is unclear • No widely accepted cut-points to identify patients with low muscularity

*CSA* cross-sectional area, *CT* computed tomography, *ICU* intensive care unit, *L3* third lumbar

## Nutritional management in critically ill subgroups

RCTs conducted to date have focused on key practice questions, but included heterogenic populations. These studies have not shown clinical benefit with nutrition interventions for reasons previously discussed though there are several patient subgroups that may still benefit from nutrition interventions. In an attempt to investigate such groups, a number of large RCTs have included pre-planned subgroup analysis (e.g. response to the intervention according to differing BMI category). However, results from these types of analyses must be interpreted with caution as the sample size may be small. Moreover, if a benefit or harm is observed in a subgroup, but the overall trial result suggests no difference, it must be considered that another subgroup hidden in the heterogeneous population may have experienced the opposite effect.

### Malnourished

The diagnosis of malnutrition in critically ill patients is challenging. Diagnostic tools, such as the widely used Subjective Global Assessment (SGA) and criteria outlined in the recent Global Leadership Initiative in Malnutrition (GLIM) recommendations, rely heavily on obtaining accurate anthropometrical data, weight and diet history, and the assessment of muscle mass, all of which are difficult to acquire in the acute early phase of ICU admission [61]. For this reason, RCT evidence attempting to investigate if patients who may be malnourished respond differently to nutrition is limited to subgroup analysis in patients with differing BMI categories or nutrition risk scores [10, 12, 67]. To date, no benefit has been observed when more or less nutrition is provided in these subgroups although the numbers included are often small. Further, BMI is a poor surrogate measure for malnutrition, and commonly used nutrition risk scores have not been well validated, which limits any conclusions on how nutrition therapy may affect outcomes in this vulnerable subgroup [2]. Despite the lack of evidence in this area, the authors support minimising progression of malnutrition. Where possible, clinicians should use local hospital guidelines or the recent GLIM criteria, combined with clinical judgement to diagnose malnutrition. As outlined in Fig. 1, in severely malnourished patients, we encourage early low dose nutrition therapy in the acute early phase, with a slow progression to target during the acute late phase, while carefully monitoring for refeeding syndrome.

### Obese

The unique and complex care needs of obese patients (BMI ≥ 30 kg/m^2^) are amplified when they become critically ill and include a greater risk of insulin resistance and loss of lean muscle mass, and wide variations in macronutrient metabolism, which makes nutrition management complex [4, 68]. There is currently very limited, low-quality evidence to inform nutrition provision in the critically ill obese patient, and as a result, the latest clinical guidelines provide inconsistent recommendations regarding energy and protein targets (Table 1).

In the TARGET trial, 1423 obese critically ill patients were included, representing the largest population of obese patients in an ICU nutrition study [8]. While not statistically significant, the obese subgroup was the only pre-specified subgroup where the point estimate sat on the side of benefit with greater energy delivery [8]. These results require formal evaluation in a robust, adequately powered and blinded clinical trial; however, they highlight that obese patients may respond differently to nutrition delivery than non-obese individuals and that there is a critical need for further research in this patient group.

In the absence of definitive evidence of the impact on functional recovery in particular, it is the opinion of the authors that obese patients should be managed like any other patient admitted to the ICU. If predictive equation estimates are used, a method to adjust body weight should be used in the nutrition prescriptions (not actual weight), and delivery monitored carefully with the knowledge that most predictive equations significantly underestimate requirements in this group [69]. It may be appropriate to consider a weight loss regime in the recovery phase once the acute illness has resolved.

### The non-ventilated patient

Critically ill patients who are not intubated may have prolonged periods of inadequate oral intake. In a prospective observational study, 50 patients who were not receiving any EN or PN were studied for 7 days following endotracheal extubation [70]. The average daily energy and protein intake failed to exceed 50% of daily requirements on all 7 days for the entire population [70]. To prevent malnutrition, it is important that clinicians monitor the oral intake of awake patients and the authors support the ESPEN guideline recommendation that medical nutrition therapy should be considered for all patients staying in the ICU for > 2 days regardless of their ventilation status [2].

## Post-ICU

The limited data available indicates that the predominant mode of nutrition following an ICU admission is via the oral route and nutrition intake in this period remains below clinician recommendations. In 32 patients from 2 centres, nutrition intake was assessed 3 times per week in the post-ICU phase [71]. Oral nutrition was the most common type of nutrition therapy (55% of study days) [71]. The median [interquartile range] energy and protein intake was 79% [41–108%] and 73% [44–98%], respectively; however, considerable variation was observed depending on the type of nutrition therapy provided, with energy and protein provision the lowest in patients who received no additional oral nutrition supplements (37% [21–66%] of target energy and 48% [13–63%] protein) [71]. A second single-centre study of patients with traumatic brain injury indicated poorer intake post-ICU compared to in ICU, and nutritional deficit was significantly greater in patients who consumed oral nutrition alone compared to those receiving artificial nutrition support [72]. Despite this, dietitians spent just 20% of their time managing patients receiving oral nutrition therapy and saw the patients a mean of 2.2 (1.0) times per week for 34 (20) min per occasion on the post-ICU ward [72]. The predominant issues impacting nutrition intake are reported as appetite, disinterest in food, and taste changes [73].

Unfortunately, non-individualised, ‘one-size fits all’ processes to the management of nutrition are likely impacting on nutrition adequacy in the post-ICU period. In one of the only studies investigating processes that impact nutrition in the post-ICU period, it was found that of nine patients transferred to the post-ICU ward, six had their gastric tube removed on the advice of the medical team without assessment of nutrition intake [73]. Early removal of gastric tubes may improve patient comfort and is encouraged by many post-surgical protocols, but has the potential to negatively impact nutrition intake [73]. The decision to remove a tube should be made on a case-by-case basis and after consultation with the patient, the treating team, and the dietitian [74]. Among other possible causes, it is plausible that inadequate nutrition following critical illness may result in significant energy and protein deficit and may explain the lack of benefit in long-term outcomes observed in nutrition studies that have delivered an intervention in the acute early and late phases. This is an important knowledge gap for investigation and to provide initial insights; a multicentre RCT is underway (ClinicalTrials.gov NCT03292237).

## Conclusion

Results from recent large-scale trials highlight that in heterogeneous groups of patients, full feeding in the acute phases of critical illness does not provide an advantage over trophic feeding and may be harmful. It remains uncertain what impact specific nutrition interventions have in the recovery phase of illness and in specific subgroups who may respond differently to nutrition interventions. The effect of nutrition delivery on other clinically meaningful outcomes, such as muscle health and physical function, is also insufficiently studied. We recommend nutrition prescriptions that tailor for pre-admission nutrition status, and severity and stage of illness. Particular attention should be paid to patients that are in (or likely to stay in) ICU for greater than a week, with ongoing monitoring of nutrition delivery and regular review of measured or estimated nutrition requirements.

## Data Availability

Not applicable.

## Abbreviations

ASPEN/SCCM: American Society of Parenteral and Enteral Nutrition/Society of Critical Care Medicine
EN: Enteral nutrition
ESPEN: European Society of Parenteral and Enteral Nutrition
ICU: Intensive care unit
PN: Parenteral nutrition
RCT: Randomised control trial
